# How informed is declared altruism in clinical trials? A qualitative interview study of patient decision-making about the QUEST trials (Quality of Life after Mastectomy and Breast Reconstruction)

**DOI:** 10.1186/s13063-016-1550-7

**Published:** 2016-09-02

**Authors:** Natalie Bidad, Lindsay MacDonald, Zoë E. Winters, Sarah J. L. Edwards, Marie Emson, Clare L. Griffin, Judith Bliss, Rob Horne

**Affiliations:** 1 Department of Practice and Policy, Centre for Behavioural Medicine, School of Pharmacy, UCL School of Pharmacy, Mezzanine Floor, BMA House, Tavistock Square, London, WC1H 9JP UK; 2 Breast Cancer Surgery Clinical and Patient Reported Outcomes Research, School of Clinical Sciences, University of Bristol, Level 2 Learning and Research, Southmead Hospital, Bristol, BS10 5NB UK; 3 Centre for Health Humanities, University College London, Gower Street, London, WC1E 6BT UK; 4 Division of Clinical Studies, The Institute of Cancer Research Clinical Trials and Statistics Unit (ICR-CTSU), London, SM2 5NG UK

**Keywords:** Breast cancer, Altruism, Informed consent, Qualitative

## Abstract

**Background:**

Randomised controlled trials (RCTs) often fail to recruit sufficient participants, despite altruism being cited as their motivation. Previous investigations of factors influencing participation decisions have been methodologically limited. This study evaluated how women weigh up different motivations after initially expressing altruism, and explored their understanding of a trial and its alternatives. The trial was the ‘Quality of Life after Mastectomy and Breast Reconstruction’ (QUEST) trial.

**Methods:**

Thirty-nine women participated in qualitative interviews 1 month post-surgery. Twenty-seven women (10 trial decliners and 17 acceptors) who spontaneously mentioned ‘altruism’ were selected for thematic analysis. Verbatim transcripts were coded independently by two researchers. Participants’ motivations to accept or decline randomisation were cross-referenced with their understanding of the QUEST trials and the process of randomisation.

**Results:**

The seven emerging themes were: (1) altruism expressed by acceptors and decliners; (2) overriding personal needs in decliners; (3) pure altruism in acceptors; (4) ‘hypothetical altruism’ amongst acceptors; (5) weak altruism amongst acceptors; (6) conditional altruism amongst acceptors; and (7) sense of duty to participate. Poor understanding of the trial rationale and its implications was also evident.

**Conclusions:**

Altruism was a motivating factor for participation in the QUEST randomised controlled trials where the main outcomes comprised quality of life and allocated treatments comprised established surgical procedures. Women’s decisions were influenced by their understanding of the trial. Both acceptors and decliners of the trial expressed ‘altruism’, but most acceptors lacked an obvious treatment preference, hoped for personal benefits regarding a treatment allocation, or did not articulate complete understanding of the trial.

**Trial registration:**

QUEST A, ISRCTN38846532; Date assigned 6 January 2010.

QUEST B, ISRCTN92581226; Date assigned 6 January 2010.

## Background

Randomised controlled designs for trials of different treatments (RCTs) are essential to minimise selection bias and ensure that treatments are based on the best possible evidence. However, many trials do not recruit the desired participant numbers required to meet study objectives, despite often citing altruism as their motivation for participating. The statistical power of the results then becomes compromised or the costs through extensions to the recruitment period are increased [1]. In order to maximise the rates of trial participation whilst ensuring patients can make their decisions based on a clear understanding of what is involved, it is imperative to understand factors affecting motivation and patient decisions around participation and non-participation in trials.

Altruism, defined as ‘acting with an unselfish regard for others’ , has been identified as a potentially important factor in patient’s decision-making as to whether or not to consent to trials. Altruistic motivations in healthcare trials include patients’ desire to help others with the same condition and contribute to progressing medical knowledge [2]. However, factors other than altruism may be motivating factors in patients’ decisions; although cancer trial participants within drug-intervention studies commonly reported that altruism contributed to their decision to enrol, they also expected to receive medical benefits [2–4].

Current studies evaluating the levels of trial participation are methodologically limited; involving either healthy participants responding to hypothetical scenarios in analogue studies; and by focusing exclusively on those patients consenting whilst ignoring those who declined [5]. Previous surveys are difficult to interpret as respondents were rarely asked to make a choice between self-interest and altruism, typically reporting both motivations without exploring how these motivations were weighted [4].

Importantly, regulation of most new drugs and devices mean that they are available to patients only if they agree to participate in a clinical trial, making it difficult to disentangle their motivations toward science and themselves. Many agree to participate in the hope of receiving experimental treatment as well as contributing to medical knowledge. Different trial designs include control arms which could be a standard treatment or a placebo (even sham surgery in some cases), which compound the difficulty in interpreting results about motivation. [6, 7]. Recently, McCann et al. [8] conducted a qualitative study, embedded in a ‘patient-preference’ trial (in which patients could either select their preferred treatment or accept random allocation of those treatments). Results suggested that people rarely participate in trials out of purely altruistic reasons, and often require some perceived personal benefit from being randomised in a trial.

Our study investigates what is often regarded as a socially desirable, yet unexplored, response of altruism. To better understand how patients weigh altruism in their decision-making about participating in clinical trials we also examined any stated personal treatment preferences and understanding of fundamental aspects of RCTs, such as randomisation and clinical equipoise. The effects of altruistic motivations on decisions about participation might be moderated by misperceptions of randomisation, equipoise or by treatment preferences [7]. For example, patients may be motivated to participate because they believe that the research will benefit others but decide against taking part because they have not understood the attributes of an RCT. On the other hand, a decision to participate in the trial might seem to be purely altruistic if the patient has a strong personal treatment preference and their decision to participate in the trial is made with a full understanding that, through randomisation, they may not receive the preferred treatment that they could have selected outside the trial.

**Fig. 1:**
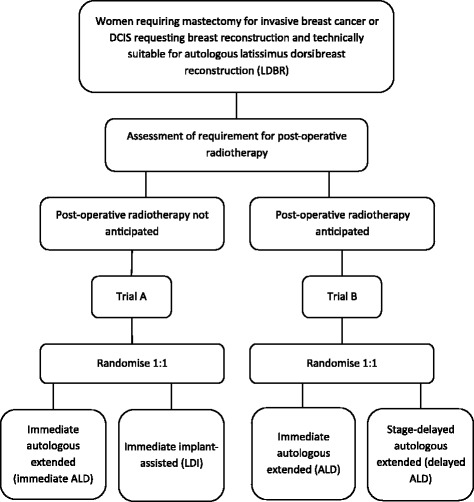
Randomisation in the QUEST trials

The Quality of Life after Mastectomy and Breast Reconstruction (QUEST) trials (Cancer Research UK funded: C10318/A10077, reference Trial A ISRCTN: 38846532, Trial B 92581226) comprised two parallel feasibility phase III randomised multicentre trials to assess the impact of the type and timing of latissimus dorsi (LD) breast reconstruction on health-related quality of life (HRQL) when post-mastectomy radiotherapy is unlikely (Trial A) or highly probable (Trial B) [9]. All women with either invasive breast cancer or ductal carcinoma in situ requiring mastectomy were eligible (Fig. 1) [9]. Surgical treatment arms comprised a standard care arm versus a less practiced new intervention arm in both trials. The standard of care was implant-assisted LD breast reconstruction in Trial A, and staged-delayed (two-stage) extended autologous LD breast reconstruction in Trial B. Despite its pragmatic design, the LD breast reconstruction techniques were well established in the UK, and were potentially available to patients outside the trial.

The QUEST Perspectives Study (QPS) was an embedded study evaluating the perceptions of patients and healthcare professionals on randomisation (views on decision-making and subsequent experiences) to inform QUEST trial processes and enhance ongoing recruitment and patient acceptability. In the current analysis, we examined patients’ views about altruism, as a factor in their decision to participate (or not) in a clinical trial, in the context of: (1) their understanding of randomisation (the rationale and trial process); (2) their comprehension of clinical equipoise related to the impact of health-related quality of life (HRQL) on the types and timings of immediate LD breast reconstruction; and (3) their perceptions of and preferences towards the treatment options available to them both before and after randomisation.

## Methods

### Recruitment and procedure

QUEST and QPS were each approved by South West Exeter Health Research Authority (QUEST Trial A 10/H0206/41, QUEST Trial B 10/H0206/42) [9]. Eligible patients approached to participate in QUEST were simultaneously invited (face to face) to participate in QPS. Informed consent was gained from all participants for both the QUEST trials and QPS. The women who consented to the QPS comprised both decliners and acceptors of QUEST. They were invited to participate in a semi-structured telephone interview 1 month after their breast reconstruction surgery with one of the researchers (NB or LM, both experienced qualitative researchers in behavioural medicine and independent from the research team conducting the QUEST trials). Interviews were audio-recorded with participants’ permission and transcribed verbatim. The interviews explored the women’s understanding of the QUEST trials, the process of randomisation and their perception of the surgical options. The interviews also explored patients’ decision-making processes in accepting or declining to enter QUEST and the factors they considered when making the decision including their eventual motivations influencing their decision.

One hundred and twenty-four patients were eligible to enter QPS, and 56 (45 %) consented, of whom 39 took part in qualitative interviews [9]. Reasons for consenting to, but not completing, the interviews included the following: researchers were unable to contact the participant after multiple attempts and changes in participants’ personal circumstances making interviews inappropriate. The analysis presented here focuses on those interviews in which participants spontaneously raised ‘altruistic’ motivations to explore the meaning and types of genuine sentiment or socially desirable answers (*n* = 27). The demographic characteristics of this subset of the interviewees are summarised in Table 1.

**Table 1:** Demographics of interviewees included in current analysis

	QUEST acceptors (*n* = 17)	QUEST decliners (*n* = 10)	Total (*n* = 27)
Age, mean (sd)	53.2 (9.28)	54.7 (10.22)	53.7 (9.37)
Ethnicity			
White British	15	10	25
White Irish	1	0	1
Black African	1	0	1
Education, n (%)			
Degree	8 (47)	5 (50)	13 (48)
Continued after minimum school-leaving age	2 (12)	3 (30)	5 (19)
Did not continue after minimum school-leaving age	7 (41)	2 (20)	9 (33)
Marital status, n (%)			
Single	3 (18)	0	3 (11)
Married/living together	13 (76)	6 (60)	19 (70)
Separated/divorced	1 (6)	3 (30)	4 (15)
Widowed	0 (0)	1(10)	1 (4)
Dependent children, n (%)	9 (53)	3 (30)	12 (44)

### Data analysis

Two authors (NB and LM) initially independently coded the transcripts using NVivo 10 software. Using the grounded theory approach, thematic analysis enabled codes to be conceptualised into common themes [10]. Emerging themes were subsequently discussed with and guided by SE (an experienced bioethicist and qualitative researcher in research ethics and independent from the QUEST trial research team). The themes were further developed by NB and LM using the original interview transcripts. After independent coding, consensus agreement was reached through discussion where initial coding differed. Participants’ expressed motivations to accept or decline the trial were the key finding.

To explore the inter-relationships between these factors, the expressed altruism was cross-referenced with other expressed motivations, and with understanding of the trial, of randomisation, and of alternative treatment options (as judged independently and agreed by NB and LM), as well as with their actual decision. Patients’ overall understanding was assessed independently by drawing on different aspects of the transcripts (NB and LM) and noting consistency of responses with known consequences of their actual decisions for treatment allocation. (A participant stating a treatment preference with self-interested motivation, but accepting randomisation, may have misunderstood information about the trial). A full analysis of understanding will be reported elsewhere. Additional findings from QPS, other than the spectrum of altruistic motivations are presented elsewhere [9, 11].

The themes regarding expressed altruism are presented below with illustrative quotes from participants.

## Results

### Altruism was initially expressed by both acceptors and decliners

Altruistic motivations were initially expressed both by women who accepted and declined the QUEST trials. They expressed a desire to help the investigators and to improve care for women with breast cancer in the future. In some cases, the altruistic decision to participate in the trial seemed to be taken with an understanding of trial processes and the desire to help others after receiving a life-changing diagnosis. Those who declined participation, however, also reported strong beliefs in the value and importance of research and of helping people (Table 2).

**Table 2:** Altruism expressed by acceptors and decliners

***[On altruism]*** *My reaction was if this is going to help other people who’ve got cancer I’m happy to do that, my primary thought. I think it depends on one’s personality and character as to would view in other people but I really like to do that so my main thing wasn’t actually of me, it was helping other people because it was such a shock when I found out I’d got it [breast cancer]. It was so shocking. I had no idea, not a smoker, not a drinker or anything. I was just so shocked and I thought, ‘Gosh if any information they can glean from me is going to help people’.*
***[On randomisation]*** *…obviously because of them randomising it, it’s neutral, isn’t it in a way. Whereas if one makes the choice people might always go for a certain option which then makes it a bit more complicated… I presume it’s [randomisation] giving more of a balanced view of what treatments are administered so that you can get a clearer picture of what the benefits are and facts and things like that.*
Trial acceptor, full understanding of randomisation (participant 7)
***[On altruism]*** *I wasn’t thinking of it so much for myself [potential trial benefits] but I think any diagnosis of a major illness changes your outlook anyway and it is such a life-changing thing that anything that I felt I could do to contribute to things being better for anyone in the future I just thought it was a good opportunity to do that and it wasn’t anything that was going to jeopardise my treatment or any outcome for me in any way but at least I thought it wouldn’t.*
***[On randomisation]*** *…the computer would choose which option and it was because that’s the fairest way to do the research so it’s not sort of weighted by any other considerations..*
Trial acceptor, full understanding of randomisation (participant 8)
***[On altruism]*** *For me, initially taking part in trials, it was simply that I worked in clinical trials and obviously working in it, it’s something I believe in and it was something I wanted to help with so for me that was why I wanted to be involved in some of them.*
***[On randomisation]*** *[Researchers used randomisation] So that the conclusions that you draw at the end of it are not influenced by any preconceived ideas from the knowledge of which streams you are randomised into.*
Trial decliner, full understanding of randomisation (participant 25)
***[On altruism]*** *Well to be honest I didn’t mind because I thought if it helps other people that I didn’t mind and I thought I agreed to take part in another study where they were taking my blood as well and stuff.*
***[On randomisation]*** *I was aware that the computer, this is how I was made to think it was whether it was right or wrong, that the computer would decide on my surgery. And I was kind of like ooh how… it doesn’t know me, do you know what I mean? Obviously, in retrospect, thinking about it all the relevant information would have been inputted into that computer, I know that now, but at the time it was no, it’s not a person. […] it wasn’t explained to me if I could disagree with the computer.*
Trial decliner, did not fully understand randomisation (participant 22)

Not all the women expressing altruistic motivations were able to articulate full understanding of the rationale for and process of randomisation, and therefore their decision whether or not to take part could not be seen as fully informed (Table 2, participant 22).

The initial expression of altruism therefore did not seem to differentiate between decliners and acceptors. Some of those who declined to participate yet expressed altruistic intentions did not fully understand what randomisation involved. However, participants’ discourse often revealed other motivating factors beyond the initial desire ‘to help others’ by taking part in QUEST. These cause us to question whether those who declined would have reached a different decision had they had a better understanding of randomisation. The factors influencing the eventual decisions by decliners and acceptors are described in the following themes.

### Personal needs took precedence in decliners

Other factors overrode the altruistic motivations initially expressed by those who declined. For most decliners, their own current needs took priority over any altruistic inclinations and, although they were interested in helping other people and accepted the importance of research (with many fully understanding both the rationale for and process of randomisation), they felt that, at this time, it was more important to focus on themselves and what was right for them (Table 3, participant 19).

**Table 3:** Personal needs taking precedence

***[Own need]*** *I don’t know, it’s just my personality thing, I’m just aware that research needs to be done, […] and I just thought ‘Oh God I ought to do it’ and then I thought ‘No, absolutely not, it’s not for me’. […] to be honest it’s the control thing because when you’re diagnosed you just feel like your life’s on hold. People are saying, ‘Can you come and do this that and the other,’ and you think, ‘Well I don’t know. I don’t know when my operation is going to be. I don’t know when the tests are going to be. I don’t even know if I’ll be able to go out at work or what.’ You’ve got no control over anything. Then to not even have any control over the process, the procedure it just seemed like yet another decision that I didn’t have really.*
***[On randomisation]*** *That if I was happy to either have an implant, or not and then it would be randomly decided whether I would get one or not. […] what happens is that your name goes in a hat more or less. […] it’s just because it makes it completely fair […]*
Trial decliner, full understanding of randomisation (participant 19)
***[Own need]*** *I’m the kind of the person that’s happy to try to do things. I was a blood donor for years. I’m happy to do it voluntarily but it’s not so much that when you’re given the information you are always thinking, it sounds selfish, but, ‘What will I get out of this?’ As much as, ‘What can I give to them?’ It’s nice if you can get a little bit out of it for yourself […]*
***[On randomisation]*** *It’s like being in a lottery, isn’t it? […] When you’re in hospital you want to feel that you’re not being treated as a lottery number, you’re being treated as an individual. It’s important because then you feel that your best interests are at heart […] doing something randomly you haven’t got the researchers, they’re not choosing which patients to use it’s the randomisation so therefore you can be going from a wider spectrum of people instead of thinking, ‘Oh well I’m going to do this study and I’ll just pick out blonde-haired blue-eyed 30-year-olds,’ or, ‘I’ll pick her because I like the colour of her hair.’ You’ve got no input at all as to why you’re picking somebody. It’s completely random and that, to me, is for a study I can understand it, but as a patient you don’t like to feel you’re in a lottery.*
Trial decliner, did not fully understand randomisation (participant 27)
***[Own need and randomisation]*** *I didn’t mind being asked, I was quite happy to go into the trial for that reason, because it’s research and it’s proving a point and hopefully all for the benefit of mankind, that didn’t worry me. Randomised I didn’t think I really understood what they meant by randomised until it was explained to me that you get whatever treatment we give you type thing, as in the different flaps that are on offer for rebuilding your boob and all that jazz. But as soon as they said implant I baulked at implant because I didn’t want an implant.*
Trial decliner, understood rationale for but not process of randomisation (participant 16)

While accepting the potential benefits of the trial to others, some women who declined to take part did so because they not only failed to see any personal advantage in participating, but also perceived randomisation to be detrimental to their individual needs (Table 3, participant 27).

Alternatively and despite there being clinical equipoise between the options offered in the QUEST trials, some decliners retained a belief that one option was better for them than the other and therefore held a treatment preference that eventually guided their decision. However, they did not fully understand the process of randomisation, which may have been too daunting, or they seemed to want to avoid one or other of the trial treatments (Table 3, participant 16).

### Pure altruism (true selflessness) in an acceptor

Many acceptors also held treatment preferences going into the trial. Only in one case was an acceptor not allocated her expressed preference during randomisation. This lady accepted randomisation regardless, apparently demonstrating pure altruism. Her immediate preference was to have the surgery completed as quickly as possible in order to progress with her life. However, from her explanation it was clear that although she discussed a computer deciding the surgical option, she also referred to the doctors choosing the option and that they were likely to know which was better due to their experience, and therefore their decision would ultimately be better than her own decision. Therefore her understanding of randomisation was inaccurate (Table 4).

**Table 4:** Pure altruism

***[On her preference]*** *I said okay I want to finish this and get out of this mess. Sometimes that’s how I say things because that’s how I think, not say it, but that’s how I think. So as a patient you always feel to finish it quickly and just go on with your life. It doesn’t work like that. The doctors know more about us, about what we are going through than ourselves. […]*
***[On randomisation]*** *The computer makes the decision I say okay I don’t make too much like oh no I just say this is what I want, I don’t want to go to implant thingy but if that’s what they choose, the doctors know more than me so I will go ahead with the decision. […] Yes and they [doctors] said because in my case because they know what they are saying and they know what happens I don’t know. So if they say this is the best for you so I will go ahead with that.*
Trial acceptor, did not fully understand randomisation (participant 9)

### ‘Hypothetical altruism’ (selfless behaviour stated but not put to the test) amongst acceptors

Of those acceptors with treatment preferences who were allocated to them during randomisation, most stated that they would have accepted the alternative had they been allocated to it, despite being able to withdraw after randomisation and select their preferred treatment, therefore demonstrating, hypothetically, a high commitment to the trial. We have therefore labelled this as ‘hypothetical altruism’.

Some participants who were allocated to their treatment preference, but claimed that they would have accepted the alternative fully comprehended the process of and rationale for randomisation (Table 5). However, one participant (participant 13) also expressed complete trust in her healthcare team and a certain belief in the ‘equivalence’ of the treatment options (as if there were evidence that the options were equally good rather than that there was currently no evidence of differences in HRQL), which could have indicated a misunderstanding about clinical equipoise and hence a therapeutic misconception associated with the trial.

**Table 5:** Hypothetical altruists with trial understanding

***[Preference]*** *Was to have as much surgery done in one go really to be honest […] because from being diagnosed, the whole treatment pathway it’s really lengthy, everyone can appreciate that. In my mind delaying the reconstruction would have just lengthened that even more […] From what I remember, I could’ve pulled out at any time from the trial anyway. I wouldn’t have wanted to and I don’t think I would have done, but I was fully aware that I could pull out at any time which was fair and I did appreciate that.*
***[On randomisation]*** *…people can be put into a system and you were randomly picked [for different surgical options] from what I believe…. It makes it a little bit more fair doesn’t it? Hopefully you get a completely different cross-section of ladies taking part, following each process really, but you’re not getting say for example, all the ladies my age doing it one way and then all the ladies of an older age doing it the other way, then of course it’s not a fair representation is it of everybody involved.*
Trial acceptor, full understanding of randomisation (participant 15)
***Participant:*** *I was told from very early on if you have a strong view and it comes back with what you don’t want you can opt out of the trial at any time […] I didn’t want an implant because I looked further into possible post-op complications and things and when it came back it was a non-implant so that was fine. I actually in the couple of days, because I went, I think it was the Thursday, and they put me in for the trial and then the randomisation came back on the Monday, so over the weekend I was thinking please don’t let it be an implant, please don’t let it be an implant. Anyway it came back and it wasn’t an implant so that was fine.*
***Interviewer:*** *What do you think you would have done if it had come back with the other option?*
***Participant:*** *I probably would have gone with it anyway because I absolutely trust the team that are looking after me. I think that was the most important thing that you trust the team that are caring for you and my surgeon was absolutely adamant that both outcomes would be as good.*
***[On randomisation]*** *In order to be able to be on the trial I had to be suitable for either a reconstruction with an implant or without an implant … and the randomisation happened in that no one person decided, a computer spit it out or somebody spit it out and it meant that it wasn’t down to my choice […] Nobody chose, it was a computer or a person picking a name out of a hat that chose rather than the surgeon and stuff so that meant that my experience wasn’t influenced by anybody.*
Trial acceptor, full understanding of randomisation (participant 13)

Most ‘hypothetical altruists’ did not demonstrate a full understanding of randomisation and may not have fully appreciated the consequences of remaining in the trial and on a treatment they did not prefer (Table 6). Another acceptor (participant 12) thought that the trial coordinators decided on her allocation based on her clinical information. She was happy to accept either option despite a preference for no implant as she trusted the surgeon’s advice that she was suitable for both options.

**Table 6:** Hypothetical altruists without full trial understanding

***Participant:*** *[…] It wouldn’t make no difference about the trials, if I was randomised for a different type and I didn’t like it, she [the nurse] said that’s no problem, she said you don’t have to go on the trials but I agreed in the end. I thought about it and I agreed. I thought yes it’s a better outcome and for people to understand more about breast cancer.*
***Interviewer:*** *So how do you think you would have felt if you would have been randomised just to have no implant [less preferred option]?*
***Participant:*** *I wouldn’t have minded. I think after going through the operation and going through the cancer, I didn’t mind either way as long as the cancer was gone and I was a shape, I was still a woman with my own flesh there somewhere […]*
***[On randomisation]*** *I thought that the computer put in my details and then have a look through it all and see what is best for me and when I thought about it afterwards they said I could change my mind at any time, I didn’t have to go through with what the computer picked but thinking about it, and I thought yes I think I would like to go along with it.*
Trial acceptor, did not fully understand randomisation (participant 11)
***Interviewer:*** *So knowing that you had this preference not to have an implant, how did you feel about being randomised then, knowing that you might have been offered the other option?*
***Participant:*** *Well, after speaking to [surgeon], he did say that both, whichever one they decided to do, and I trust that man absolutely, that it would be perfect for me.*
***Interviewer:*** *So if you had been randomised to have an implant, you would have been happy to go along with that?*
***Participant:*** *Yes.*
***[On randomisation]*** *I think they [the Quest people] just looked at everything, what I was like, all my notes and everything and then decided. […] Well, the Quest people. […] This is how I understood it, they wanted equal amounts of women to go for and against the two procedures.*
Trial acceptor, did not fully understand randomisation (participant 12)

### Weak altruism amongst acceptors

Some acceptors did not have a treatment preference going into the trial and therefore demonstrated what could be described as a ‘weak’ form of altruism since there was an absence of personal benefit rather than an active intention to benefit others. Some of these participants had full understanding of randomisation and so their altruistic motivations were based on the knowledge that they could have been assigned to either treatment at random (Table 7).

**Table 7:** Weak altruism

***[On randomisation and no preference]*** *[…] if you were suitable, you could be randomised, i.e. the computer would decide which operation you were having and the outcomes were both, I was explained that there were pros and cons to both and I was happy to have both of the surgeries and actually it was quite nice for me because I don’t think I could have decided […] obviously on the […] study makes it fairer. If they’d picked me to do something, it could’ve been for a reason, it could’ve been for something I said whereas letting a computer decide is completely random and it chooses what it likes based on nothing whatsoever.*
Trial acceptor, full understanding of randomisation (participant 5)

### Conditional altruism (selflessness dependent on perceived personal benefits) amongst acceptors

For some acceptors, their altruistic behaviour appeared to be contingent on perceived immediate personal benefits from taking part in the trial, recognised elsewhere and termed conditional altruism [8]. Expanding on the weak altruism presented above, for some women, not having to choose was a direct benefit of participating in the trial. They reported that decision-making was extremely difficult either because they had no preference or because they were overwhelmed by their diagnosis and the quantity of information. The first participant demonstrated full understanding of randomisation, the second understood the rationale, but did not articulate an understanding of how it was conducted (Table 8).

**Table 8:** Conditional altruism

***[Choice made]*** *[…] as I say it’s the least I can do and also I feel quite chosen to be one and it’s helped me which you know I don’t know whether with QUEST when you set it up thought, oh they might feel a little comfort from this; my comfort is that I didn’t have to decide what I was having at a time when I was making decisions.*
***[On randomisation]*** *I know that that was okay and I could understand why they said you can’t choose the computer has to choose for you and I could see that, because you wouldn’t get the fair result would you if we all started to say “we’ve got to choose one”.*
Trial acceptor, full understanding of randomisation (participant 1)
***[Choice made]*** *I think it was just having the choice made for me and as I say I thought well if any data or research that would be useful that would come out of it then if it helps other people later on down the line then I am quite happy to do that.*
***[On randomisation]*** *I think they are obviously trying to find which surgery is best for most people […] if you are in the position where you have got two options of surgery I think they are obviously, I thought they were trying to find out which was really best for patients in the long run, collecting data on the two different procedures because I suppose if a surgeon is choosing or a patient is choosing you could only get one type of surgery being done far more than the other and perhaps they want to find out, make it something a bit fairer really and see how patients respond to the different types of operation and surgery.*
Trial acceptor, understood rationale for but not process of randomisation (participant 2)

One acceptor regarded consenting to randomisation as a way of regaining personal control over her diagnosis (Table 9, participant 5). She demonstrated full understanding of randomisation and was in clinical equipoise as illustrated in the weak altruism theme above. Another perceived advantage for participants was that they felt they would gain more attention and be better listened to as part of the trial (Table 9). No such benefits were highlighted to participants during the recruitment process, yet participants could have perceived the additional contact time as being beneficial. In addition, the focus of the trial was quality of life outcomes after surgery, and therefore participants may have perceived that in participating there may be an increased emphasis on this. However, as illustrated by participants’ quotes in the themes previously discussed, these participants understood that the best surgical option for them was decided upon through their clinical information. Both expressed a preference for their surgery but would have been happy to accept the alternative due to their belief in the surgeon’s view that the other option was equally suitable for them clinically.

**Table 9:** Perceived trial benefits or advantages

*It was a positive for me, it was something I could do that was a positive from it all basically. I think lots of things that happen to you when you’re having surgery and going through this procedure, you’ve got no control over, but actually I could choose what I wanted to do. I could feel that there was some positive coming out of it whatever the outcome was for me, which was quite nice.*
Trial acceptor, full understanding of randomisation (participant 5)
*Yes, I do actually because, like yourself ringing me now, because I thought someone would ring in a month and then three and so on. You're getting to know how I'm feeling to help other people. And, also, you're keeping checks on how we're doing which I think is a brilliant thing, more so than if I wasn't doing the QUEST.*
Trial acceptor, did not fully understand randomisation (participant 12)
*I thought I’d get the best care because they’re [QUEST investigators] really interested in what you’ve got to say and how you feel and I thought well, this way and also that other women could benefit by it as well.*
Trial acceptor, did not fully understand randomisation (participant 11)

### Sense of duty to take part

Some acceptors reported participating in the trial out of a sense of duty instead of expressing straightforward altruistic motivations in their reasons for wanting to take part (Table 10). They felt that by taking part they would be able to ‘pay back’ for all the treatment they had received and for the contributions that women before them had made to enable them to have the treatment they had. While most of these women had full understanding of randomisation as in the two quotes below, some did not and this misperception was that the most suitable treatment option would be selected based on their clinical information. Consequently, they did not perceive any personal sacrifice through participating in research. Two women who indicated this ‘sense of duty’ to participate had clear treatment preferences, but nevertheless consented to randomisation. One was allocated her preference and one was not and both indicated that they would have accepted the alternative as described in the previous sections.

**Table 10:** Sense of duty

*It seemed quite straightforward so they [healthcare team] were very, very good at the whole thing, and I just thought well, this is the least I can do you know, it wasn’t hard to decide.*
Trial acceptor, full understanding of randomisation (participant 1)
*They’re important really [trials] because my treatment from start to finish has been influenced by other ladies going through similar trials, for all sorts of different reasons. Really, it was me putting my little bit back in for women in the future to be honest.*
Trial acceptor, full understanding of randomisation (participant 15)

## Discussion

This study explores the meaning of expressed altruism when patients are invited to participate in a surgical RCT where the primary outcome measure comprised HRQL and where the new surgical interventions were available outside the trial. This study illustrates how patients weighed up different motivations, after initially expressing altruism, to decide whether or not to accept the trial, and reports how informed those motivations were, given the patients’ understanding of the trial and the treatment options available, and any stated personal preferences.

Altruism was expressed initially by both acceptors and decliners of the QUEST trials. These initial motivations were often modified by understanding the trial and individual beliefs about the surgical options, and, in many cases, were overridden by personal interest according to treatment preference. Moreover, some acceptors who were in ‘clinical equipoise’ seemed simply not to mind being randomised. Altruism was still expressed by some who had accepted randomisation despite having a treatment preference. All but one of these patients were randomly allocated to their treatment preference and so did not have to ‘test’ their declared altruism afterwards by potentially withdrawing after randomisation.

Our findings somewhat corroborate results from a recent qualitative study suggesting that altruistic motivations amongst those who accept randomisation are often tempered by self-interested motivations in various ways even when they are in a state of clinical equipoise [8]. However, it was unclear how informed these motivations were. Some patients perceived benefits of taking part in the QUEST trials, such as avoidance of making a difficult choice between surgical options, gaining an element of control by deciding to participate or perceived greater input from the healthcare team, and their altruistic motivations were conditional upon this. There is some, albeit weak evidence from comparative studies suggesting that patients may do better within trials on similar treatment to those outside of trials [12]. This study further explores conditional altruistic motivations by cross-referencing the patient's declared motivations with their actual decisions and their understanding of the trial. Only one patient in our study consented against her declared treatment preference, but she did not fully understand the trial and it was unclear whether her decision to take part was an authentic choice to be altruistic (i.e. one that reflects the true preference of the individual).

Our finding that patients did not always fully understand the trial and its alternatives (both acceptors and decliners) concurs with previous work [13]. There appeared to be misunderstanding of how the treatment option was decided if they entered the trial and, related to this, some participants did not hold views consistent with ‘clinical equipoise’. For decliners, misunderstanding randomisation and equipoise may not be the sole factors responsible for them choosing not to take part as they had strong preferences for a particular surgical option or they wished to make the decision by having control themselves. Findings from this study were regularly reported to the QUEST Trial Management group and the Trial Steering and Data Monitoring Committee whilst the trials were running, and helped inform ongoing recruitment strategies. A standardised trial information checklist was an early by-product of this study 3 months after trial commencement. The use of this checklist by the research nurse within trial consultations was standardised across all centres and was instrumental in attempting to balance expressed patient preferences [9].

We did not include all those respondents in QPS (namely those who did not express any altruism) in our present analysis because there would have been no way of weighing up different motivating factors in the unique way QUEST allowed. While an expression of self-interest alone does not necessarily exclude all altruistic motivation, we were primarily interested in those situations in which self-interest and altruism led to contradictory decisions to help gauge the strength of those motivations.

The challenges of surgical trials are well described by Cook et al. (2015) with recommended trial designs including a feasibility phase and embedding qualitative research to inform trial processes [14]. As the first multicentre trials in this setting, the QUEST feasibility trials showed overall patient acceptability rates of 19 % (17 out of 88) in Trial A and 22 % (8 out of 36) in Trial B, respectively over 18 months of recruitment [9].

The findings from this study should be considered in light of its limitations. The interviews were conducted after surgery, and at least a month after the women had made the decision whether or not to accept randomisation. In addition to potential difficulties with accurate recall, it is possible that initial preferences and perceptions were modified as a result of the outcome of randomisation (for acceptors) and post-surgery experiences. Furthermore, the QUEST trials were surgical RCTs, comparing the impact on HRQL of different types and timing of LD breast reconstruction. In addition, the treatments offered in the trial, while available outside the trial, may not always have been offered to patients making the trial a ‘quasi-preference’ trial. The findings from the current study may not be generalizable to other RCTs attempting to recruit from different patient populations, or to evaluate the efficacy of a novel drug treatment against either an active or placebo control, and under different conditions.

Nevertheless, the knowledge gained from this study provides valuable insight that can inform and refine attempts to recruit participants to future RCTs. In particular, information given to patients at the time of consent should clarify the potential benefits of trial participation. The dedicated trial consultation should be led by non-biased healthcare professionals using standardised informed consent where iteration of pros and cons may change/balance patient preferences and perceptions. Evaluation of the patient’s understanding of information as this pertains to randomisation should be routinely assessed to ensure that any expressed altruism is based on truly informed consent. In addition, more research is needed in this area to explore the limits to conditional altruism once patient understanding of trials has been improved in practice. For example, it is not yet clear how perceiving beneficiaries of the trial influences feelings of altruism. Currently, patients are often simply told - vaguely - that the trials to which they are invited to participate in, are designed to benefit patients in the future. This futuristic expectation risks undermining the fundamental precepts of good informed consent, where the patients’ understanding of the rationale and benefits of trial participation are key goals in current trial recruitment. Recommended testing of patient’s understanding of informed consent should be an integral part of feasibility and pilot phase studies within RCTs.

## Conclusions

This study makes a valuable and unique contribution to the understanding of the factors influencing patients’ decision whether or not to enter a surgical RCT where the treatment options may be preference-sensitive, despite being technically established and poorly evidenced in terms of patient-reported HRQL. By employing qualitative methods and thereby avoiding some of the limitations of previous research in this area, we have highlighted how different motivations, including altruism, were evaluated by the participants in the context of their understanding of the trial and randomisation. Although both acceptors and decliners of the QUEST trials initially expressed altruistic motivations, these were often revised or outweighed by other factors related to the participants’ own interests and perceptions.

## Abbreviations

HRQL: Health-related quality of life
LD: Latissimus dorsi
QPS: QUEST Perspectives Study
QUEST: Quality of Life after Mastectomy and Breast Reconstruction
RCT: Randomised controlled trial
